# Molecular basis for integrin adhesion receptor binding to p21-activated kinase 4 (PAK4)

**DOI:** 10.1038/s42003-022-04157-3

**Published:** 2022-11-17

**Authors:** Byung Hak Ha, Sezin Yigit, Nalini Natarajan, Elizabeth M. Morse, David A. Calderwood, Titus J. Boggon

**Affiliations:** 1 The Department of Pharmacology, Yale University, 333 Cedar St., New Haven, CT 06520 USA; 2 The Department of Cell Biology, Yale University, 333 Cedar St., New Haven, CT 06520 USA; 3 The Department of Molecular Biophysics and Biochemistry, Yale University, 333 Cedar St., New Haven, CT 06520 USA

**Keywords:** X-ray crystallography, Kinases

## Abstract

Integrin adhesion receptors provide links between extracellular ligands and cytoplasmic signaling. Multiple kinases have been found to directly engage with integrin β tails, but the molecular basis for these interactions remain unknown. Here, we assess the interaction between the kinase domain of p21-activated kinase 4 (PAK4) and the cytoplasmic tail of integrin β5. We determine three crystal structures of PAK4-β5 integrin complexes and identify the PAK-binding site. This is a region in the membrane-proximal half of the β5 tail and confirmed by site-directed mutagenesis. The β5 tail engages the kinase substrate-binding groove and positions the non-phosphorylatable integrin residue Glu767 at the phosphoacceptor site. Consistent with this, integrin β5 is poorly phosphorylated by PAK4, and in keeping with its ability to occlude the substrate-binding site, weakly inhibits kinase activity. These findings demonstrate the molecular basis for β5 integrin-PAK4 interactions but suggest modifications in understanding the potential cellular role of this interaction.

## Abstract (web summary)

Crystal structures of integrin β5 tails with PAK4 reveal a unique interaction in which integrin β5 is not phosphorylated by PAK4 but rather inhibits PAK4 activity.

## Introduction

Integrin adhesion receptors are the major mediators of cell-substratum adhesion and play vital roles in control of cell morphology, migration, and differentiation^1–4^. Importantly, by binding extracellular ligands via their complex multidomain extracellular portions and associating with intracellular signaling scaffolds, cytoskeletal proteins, and enzymes through their short cytoplasmic tails, these heterodimeric glycoproteins transmit mechanical force and biochemical signals bi-directionally across the plasma membrane^5–7^. Inside-out signaling involves the interaction of the integrin β-subunit cytoplasmic tail with proteins that increase (e.g. talin, kindlin) or reduce (e.g. filamin, ICAP1) the affinity of the integrin for extracellular ligands^8,9^; the tools of structural biology have proven to be central to understanding how these effects take place and for defining the mechanics of inside-out signaling at the molecular level^10–15^. Outside-in signaling has been shown to involve extracellular ligand-triggered activation of intracellular signaling cascades largely through the interactions of integrin β tails with cytoplasmic signaling and adaptor protein networks^16,17^. Structural biology techniques have been used to study the mechanisms of these actions, but the basis for outside-in signaling remains less well understood than inside-out signaling^18–20^.

The p21-activated kinase (PAK) group of serine/threonine kinases plays important roles in cell adhesion, motility, growth, and survival^21–24^. These kinases are functionally under the control of Rho-family small GTPases, although the molecular basis of regulation differs among the group^25–29^, and their activity is thought to be impacted by other binding partners^25,30,31^. In addition to their roles as enzymes, PAKs also modulate signaling by acting as adaptor proteins^32,33^. Thus, similar to integrins, PAK function is influenced by an array of partner proteins. Notably, interactions between the β5 integrin cytoplasmic tail and the PAK4 kinase domain have been detected in yeast 2-hybrid assays, by pull-down with recombinant β5 tails and by co-immunoprecipitation^34^. This binding has been implicated in adhesion-mediated control of PAK4 activity and in cell motility^35,36^ but mechanistic insights have been limited due to a lack of detailed information on the interaction.

Integrin function can be modulated by their cytoplasmic tails interacting with, and being phosphorylated by, protein kinases^37,38^. Studies of PAK4 suggest direct binding to the integrin β5 cytoplasmic tail^34^ followed by targeted phosphorylation of its serine residues^35^. Similarly, the nonreceptor tyrosine kinase, Arg, was shown to directly bind to the cytoplasmic tail of the β1 integrin leading to Arg-mediated β1 tail tyrosine phosphorylation^39^. In contrast, non-catalytic interactions between integrin β1 tails and the pseudokinase domain of ILK have been implicated in phosphorylation-independent control of integrin signaling^40–42^, suggesting that non-catalytic binding between integrin receptors and kinase domains has the potential to provide additional non-canonical modes of signal modulation for both integrins and kinases. These examples represent different potential mechanisms of integrin tail-kinase interaction, but until now these interactions have resisted study at the molecular level. Therefore, we used a structure-directed approach to probe the interactions of integrin β5 tail with PAK4. We determined an array of crystal structures and observe an unexpected mode of kinase-binding partner interaction; we find that the integrin β5 cytoplasmic tail engages the substrate-binding groove of PAK4 in an unusual manner that does not allow integrin phosphorylation and inhibits PAK4 activity against other substrates, resolving the molecular basis for how PAK4 can interact with integrin receptors.

## Results

### PAK4 kinase domain directly binds integrin β5 tail

**Fig. 1:**
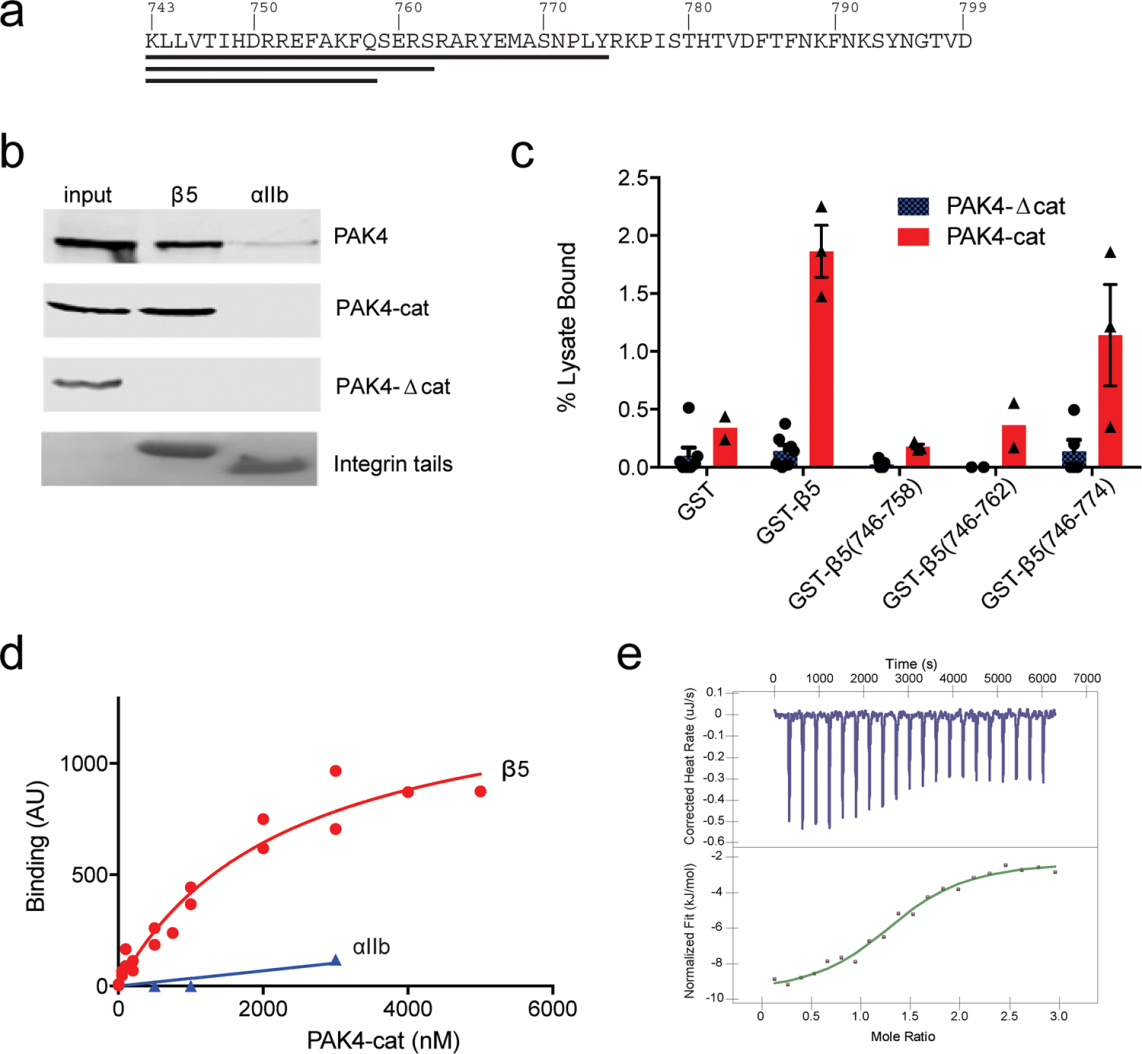
PAK4 interacts specifically with integrin β5. **a** Sequence of the integrin β5 cytoplasmic tail. Black lines indicate truncated constructs. **b** Pull down of overexpressed GFP-PAK4 constructs by nickel bead bound integrin cytoplasmic tails. Pull-down is followed by immunoblotting with anti-GFP antibodies. 3% of input lysate shown in input lane. Tail loading is assessed by Coomassie staining. **c** Mapping PAK4-binding site in integrin β5 tails. Quantification of pull-down assays for GFP-PAK4-Δcat or GFP-PAK4-cat binding to GST, or GST β5 truncations. Error bars indicate SEM. **d** Apparent affinity of PAK4 kinase domain for integrin β5 cytoplasmic tail compared to control integrin αIIb cytoplasmic tail. Results from three independent experiments were fit to a one-site specific-binding model in Prism. **e** Example isotherm for β5 cytoplasmic tail titrated into PAK4 kinase domain. Source data are provided as a Source Data file.

To probe the interactions of integrin β5 cytoplasmic tail (Fig. 1a) with PAK4 we used well-validated His-tagged recombinant models of integrin β tails^43–45^ to pull-down GFP-tagged PAK4 from CHO cell lysates. We tested binding of the longest PAK4 isoform (isoform 1) (GFP-PAK4), a construct encoding just the kinase domain (GFP-PAK4-cat, residues 302–591), and a construct encoding the N-terminal regions of PAK4 but not the kinase domain (GFP-PAK4-Δcat, residues 1–301). Both full-length PAK4 and PAK4 kinase domain bind the β5 tail but the N-terminal construct does not (Fig. 1b). This is consistent with prior reports in which the kinase domain is responsible for binding integrin β5^34,35^. Our control experiments demonstrated specificity because PAK4 does not bind to αIIb tails (Fig. 1b) and β5 tails do not bind GFP alone (not shown).

To map the binding site on integrin β5 we generated GST-fusion constructs encompassing different ranges of integrin β5 to pull down GFP-PAK4-cat or GFP-PAK4-Δcat expressed in whole cells. We find that the GST-tagged full-length integrin β5 tail (residues 743–799) binds GFP-PAK4-cat, but does not interact with GFP-PAK4-Δcat (Fig. 1c). We still observe binding with a construct that extends to residue 774, C-terminal to the previously identified S^759^ERS motif^35^, but constructs which were further truncated are strongly impaired in binding (Fig. 1c). We next established that integrin β5-PAK4 interactions are direct using His-tagged β5 tails on beads to pull down purified recombinant PAK4-cat^25^. By varying concentrations of PAK4-cat in the pull downs we calculate a *K*_d_ of ~1.8 ± 0.5 µM for the PAK4-cat interaction with integrin β5 (Fig. 1d). To assess this binding in a more quantitative manner we conducted isothermal titration calorimetry (ITC). On titration of purified integrin β5 cytoplasmic tail (residues 743–799) into purified PAK4-cat, we observe binding properties of *K*_d_ of 4.95 ± 1.5 µM, *N* of 1.05 ± 0.35, Δ*H* of −8.45 ± 0.75 kJ/mol, and Δ*S* of 73.25 ± 2.45 J/molK (Fig. 1e and Supplementary Table 1). These results strongly suggest that the catalytic domain of PAK4 directly interacts with the integrin β5 cytoplasmic tail in biochemical and biophysical experimental settings, and that the interaction includes some of the residues between Arg763 and Tyr774.

### Crystal structure of a chimeric integrin β5-PAK4-cat

**Fig. 2:**
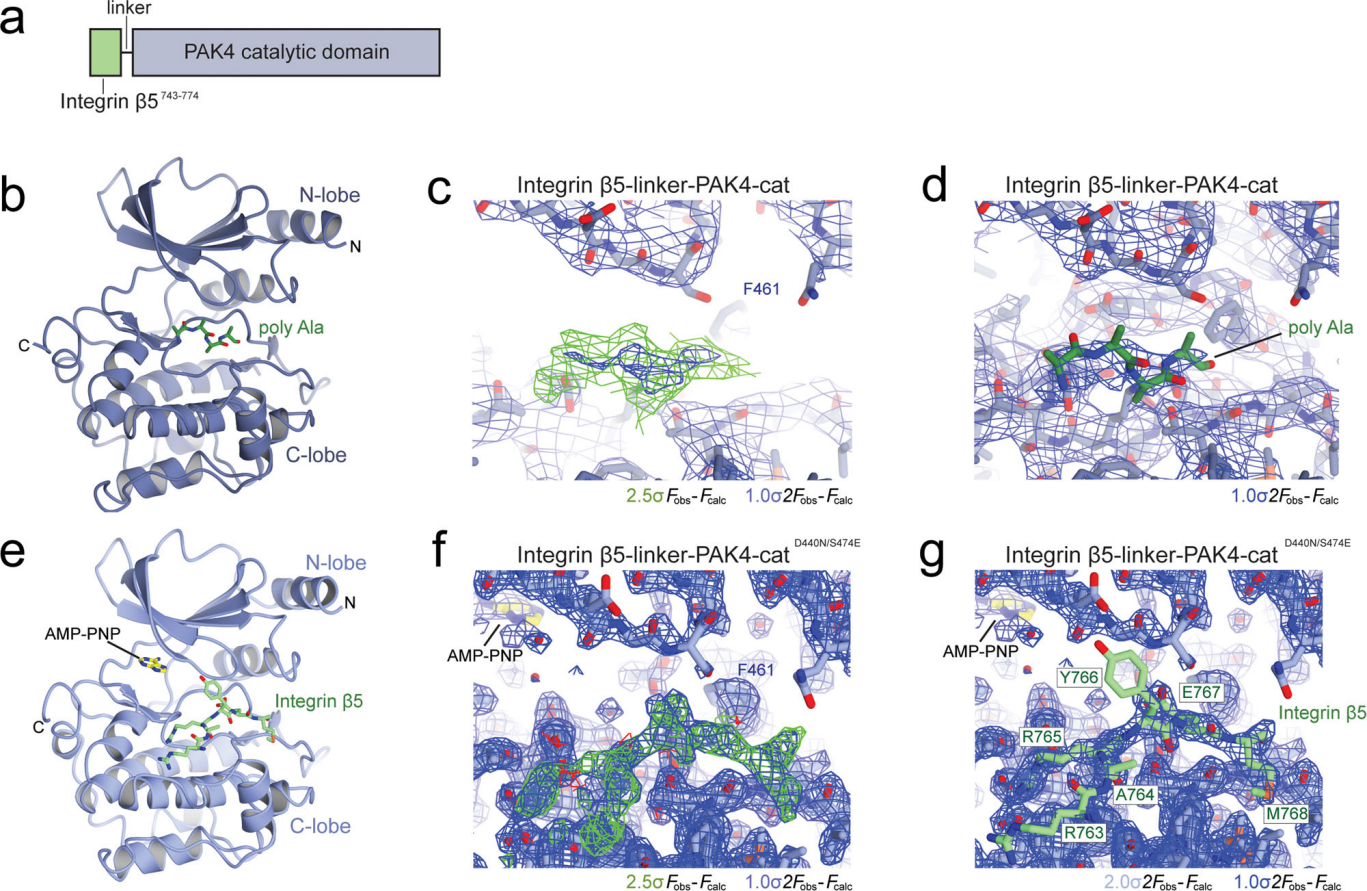
Structure determination of integrin β5 chimera with PAK4. **a** Schematic of chimeric integrin β5-PAK4. The Linker region is a 12-residue -Ser-Ser-Gly- repeat. **b**–**d** *P*6_3_ structure of integrin β5-PAK4 chimera at 3.5 Å (not submitted to PDB). **e**–**g** *P*4_1_2_1_2 structure of integrin β5-PAK4^D440N/S474E^ chimera (PDB ID: 7S47). Panels **c** and **f** show electron density maps that are unbiased by integrin β5, i.e. prior to adding integrin β5 to the model. Panels **d** and **g** show the final refined maps. *F*_obs_–*F*_calc_ maps contoured at +2.5σ (green) and −2.5σ (red). 2*F*_obs_–*F*_calc_ maps contoured at +1σ (blue) and +2σ (light blue).

To understand the molecular level details of the integrin β5 interaction with PAK4, we conducted X-ray crystallography. We did this in two phases. We began by designing chimeric constructs where the β5 tail (residues 743–774) is covalently attached to PAK4-cat via a 12-residue -Gly-Ser-Ser- repeat (Fig. 2a) that we hoped would facilitate crystallization of the integrin-bound complex. This yielded a crystal form that diffracted to 3.5 Å resolution (Table 1) and revealed that the peptide was bound but did not allow us to identify the register of the bound peptide, so we built the peptide as a poly-alanine chain (Fig. 2b–d). We then attempted to stabilize the interaction between PAK4 and integrin β5. To do this we introduced a kinase-inactivating point mutation, D440N, and an activation loop phosphomimetic mutation, S474E. These modifications allowed us to obtain a crystal form that diffracts to 2.0 Å resolution (Table 1). The electron density for the integrin β5 tail was clear and we could easily build a seven-residue region -Ser^762^-Arg-Ala-Arg-Tyr-Glu-Met^768^- into its correct register (Fig. 2e–g and Supplementary Fig. 1). This structure of chimeric integrin β5–linker–PAK4-cat provides the initial glimpse of the molecular basis for kinase recruitment to integrin cytoplasmic tails. However, chimeric crystal structures potentially induce conformational states that may not be physiologically relevant; therefore, we continued with our structural assessment of the interaction.

**Table 1:** Data collection and refinement statistics.

	Integrin β5^743–774^-linker-PAK4cat	Integrin β5^743–774^-linker-PAK4cat^D440N,S474E^	PAK4cat:Integrin β5^760–770^	PAK4cat^D440N,S474E^:Integrin β5^760–770^
		PDB ID: 7S47	PDB ID: 7S48	PDB ID: 7S46
*Data collection*				
Space group	*P6*_*3*_	*P4*_*1*_*2*_*1*_*2*	*P4*_*1*_*2*_*1*_*2*	*P4*_*1*_*2*_*1*_*2*
X-ray source and detector	APS 24-ID-E	APS 24-ID-E	APS 24-ID-E	APS 24-ID-E
	ADSC Q315	ADSC Q315	EIGER 16 M	EIGER 16 M
Wavelength (Å)	0.97918	0.97918	0.97918	0.97918
Unit cell				
*a*, *b*, *c* (Å)	142.8, 142.8, 62.3	62.3, 62.3, 178.6	61.7, 61.7, 180.0	61.8, 61.8, 178.8
*α*, *β*, *γ* (°)	90, 90, 120	90, 90, 90	90, 90, 90	90, 90, 90
Resolution				
Range^a^ (Å)	50.0–3.5 (3.63–3.5)	50.0–2.0 (2.07–2.0)	50.0–1.9 (1.97–1.9)	50–2.1 (2.18–2.1)
No. of unique reflections	9482	24727	28444	21270
Completeness^a^ (%)	100 (100)	99.8 (100)	99.4 (99.7)	99.9 (100)
*R*_pim_^a^ (%)	10.1 (651.5)	5.9 (73.7)	2.2 (28.8)	4.0 (45.7)
CC_0.5_ (%) (in high resolution bin)	41.4	61.2	77.5	44.7
Mean <*I*>/<σ*I*>^a^	8.8 (1.4)	16.3 (2.2)	26.9 (1.9)	17.5 (1.3)
Wilson *B*-factor	94.2	30.7	34.3	43.2
Redundancy	14.3 (13.5)	8.8 (8.9)	11.6 (12.5)	11.4 (9.3)
*Refinement statistics*				
Resolution range^a^ (Å)	50–3.5 (3.59–3.5)	50–2.0 (2.05–2.0)	50–1.9 (1.95–1.9)	50–2.1 (2.15–2.1)
*R*_factor_ (%)^a^	21.1 (37.7)	19.4 (27.1)	17.9 (27.9)	18.0 (30.7)
Free *R*_factor_ (%)^a^	24.0 (28.6)	23.7 (33.3)	22.2 (31.1)	22.0 (37.7)
Free *R* reflections^a^ (%)	5.4 (3.7)	5.1 (5.4)	5.2 (5.7)	4.9 (5.5)
Free *R* reflections^a^, no.	503 (24)	1258 (90)	1475 (110)	1033 (79)
Residues built				
PAK4	A/300-590	A/300–589	A/300–589	A/300–589
Integrin β5 tail	B/poly-Ala	B/763–768	B/762–768	B/762–770
No. water molecules	–	103	119	67
Mean *B*-factor (Å^2^)				
Protein (A)	99.4	25.1	30.8	35.7
AMP-PNP (A)	–	56.3	–	55.5
Protein (B)	97.2	45.7	85.3	79.6
H_2_O	–	39.7	43.8	46.0
*Model statistics*				
RMSD bond lengths (Å)	0.007	0.015	0.013	0.015
RMSD bond angles (°)	1.141	1.682	1.629	1.832
Ramachandran plot (%) favored/allowed/disallowed	94.1/5.9/0	98.6/1.4/0	97.9/2.1/0	97.6/2.4/0

^a^Indicates high resolution shell.

### Co-crystal structures of integrin β5 peptide with PAK4-cat

**Fig. 3:**
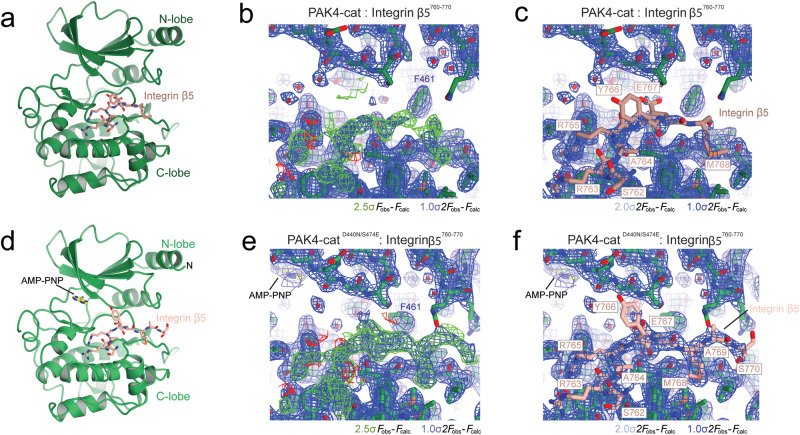
Co-crystal structure of integrin β5 peptide with PAK4. **a**–**c** *P*4_1_2_1_2 structure of wild-type PAK4 co-crystallized with integrin β5 peptide -Glu^760^-Arg-Ser-Arg-Ala-Arg-Tyr-Glu-Met-Ala-Ser^770^- (PDB ID: 7S48). **d**–**f** *P*4_1_2_1_2 structure of D440N/S474E PAK4 co-crystallized with integrin β5 peptide -Glu^760^-Arg-Ser-Arg-Ala-Arg-Tyr-Glu-Met-Ala-Ser^770^- (PDB ID: 7S46). Panels **b** and **e** show electron density maps that are unbiased by integrin β5, i.e. prior to adding integrin β5 to the model. Panels **c** and **f** show the final refined maps. *F*_obs_–*F*_calc_ maps contoured at +2.5σ (green) and −2.5σ (red). 2*F*_obs_–*F*_calc_ maps contoured at +1σ (blue) and +2σ (light blue).

To mitigate concerns of chimera-induced crystal packing, we next conducted co-crystallography of both wild-type and D440N/S474E PAK4 kinase domain with a synthesized peptide corresponding to integrin β5 residues Glu760-Ser770. We designed the peptide to encompass all of the residues we defined in the chimeric structure as interacting with PAK4, and also to include the previously identified SERS motif. Both co-crystallization experiments resulted in well diffracting crystals, to 1.9 and 2.1 Å resolution (Table 1) and provide unambiguous electron density for the bound integrin β5 peptide (Supplementary Fig. 1), which allowed us to build the peptide into its correct register (Fig. 3). For the wild-type structure we built the seven-residue region of integrin β5 -Ser^762^-Arg-Ala-Arg-Tyr-Glu-Met^768^- and for the D440N/S474E structure we built the nine-residue region of integrin β5 -Ser^762^-Arg-Ala-Arg-Tyr-Glu-Met-Ala-Ser^770^-.

### Analysis of the structures of integrin β5 in complex with PAK4

In the crystal structures, PAK4 is found in an active state with the activation loop extended away from the catalytic cleft, and the DFG motif in a DFG-in orientation. The conformation of PAK4 is very similar to previously determined structures of PAK4 in the same space groups^25,30,46–48^. In all three structures, easily interpretable electron density for integrin β5 was found in the substrate-binding groove of PAK4 kinase domain.

**Fig. 4:**
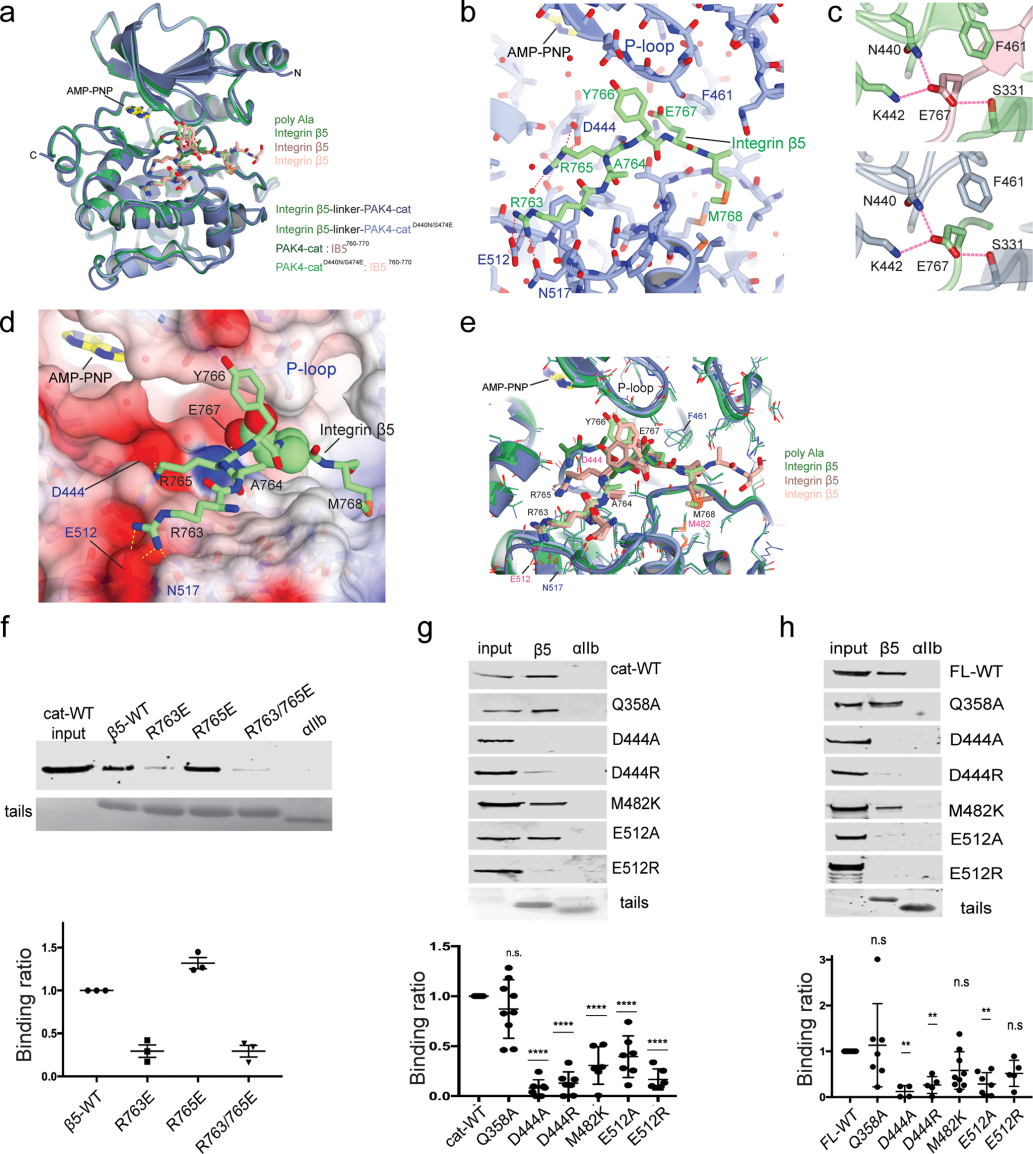
Analysis of integrin β5 interaction with PAK4. **a** Cartoon diagram of PAK4-integrin β5 structures superposed on the kinase C-lobes. **b** Details of integrin β5-PAK4 interaction. Salt bridges indicated by dashed red lines. **c** Illustration of the pose of Glu767 in the D440N mutant structures (top, bottom) Glu767 coordinates N440, K442, and Ser331. **d** Surface electrostatics of PAK4 with bound integrin β5 in green. Glu767^β5^ is located at the phosphoacceptor residue site. **e** Close up superposition of the integrin binding site. Residues mutated in panels **f**–**h** are indicated. **f** Pull down of overexpressed GFP-tagged PAK4 catalytic domain with recombinant purified wild-type and mutated integrin β5 tails immobilized on nickel beads. Bound protein was detected by immunoblotting with anti-GFP antibodies. 3% of input lysate is shown in input lane. Tail loading was assessed by Coomassie staining. A representative experiment is shown in the upper panel with the quantification of three independent replicates (normalized to the wild-type β5 tail) shown below. Mean ± s.d. Pull-down of wild-type and mutated PAK4 catalytic domain (**g**) and full-length PAK4 (**h**) with wild-type integrin β5 was assessed as in (**f**). Representative experiments are shown in upper panels and the quantification of multiple independent replicates normalized to the wild-type PAK construct are shown below. Mean ± s.d. (*n* ≥ 4). Significant difference from wild-type control calculated in one-way ANOVA with Dunnett’s multiple comparison test. Source data are provided as a Source Data file.

The three high-resolution structures are conformationally similar and crystallize in the same crystal form. Comparison of root-mean-square deviations between the three kinase domains shows deviations between 0.2 and 1.1 Å over 290 carbon alphas. Likewise, the backbones of the integrin tails are experimentally identical in conformation, with RMSDs of 0.14–0.49 Å across 6 or 7 carbon alphas (Supplementary Tables 2 and 3). The comparison of these three structures suggests that neither the chimeric nor mutational modifications to PAK4 significantly alter the overall conformation of its interaction with integrin β5 (Fig. 4a, b).

We observe differences in the mode of binding for the side chain of integrin β5 residue Glu767 bound to wild-type PAK4 compared to D440N PAK4. We find that in the D440N complexes, electron density for integrin β5 residue Glu767 is clearly visible (Figs. 2, 3 and S1) and that this hydrogen-bonds to Asn440, Lys442, and Ser331 of the glycine-rich loop (Fig. 4c). However, in the wild-type complex we observe poor electron density for Glu767 and higher overall B-factors for the peptide and Glu767. Nonetheless, it is located, albeit with lower certainty, in a similar location to the D440N structures but the DFG phenylalanine, Phe461, is rotated to accommodate binding. These findings suggest that Glu767 is accommodated in the catalytic site of both wild-type and D440N mutant PAK4.

The main driver of recognition of integrin β5 by PAK4 seems to be two integrin arginine residues, (Arg763^β5^ and Arg 765^β5^) that make salt bridge interactions with acidic patches within the kinase C-lobe (Fig. 4b, d). Further van der Waals interactions to the kinase domain are made by the integrin’s hydrophobic side chains (Ala764^β5^, Met768^β5^) (Fig. 4b, d). Overall, the orientation of the integrin peptide bound to PAK4 is similar to previous structures of PAK4 in complex with substrates^47,48^ and pseudosubstrates^25,30^. For all of these complexes, salt bridge interactions seemingly provide a major component of the interaction.

In the integrin β5-PAK4 structures, however, an unexpected conformation was observed for integrin residue Glu767^β5^. This residue is located exactly where the phosphoacceptor residue is found in the kinase-substrate structures^47,48^ and extends towards the catalytic cleft. It is surprising to observe an acidic side chain in such an orientation, and to our knowledge this has not been observed previously in kinase-substrate pairs, although a recent study of CK1δ showed binding of a phosphoserine residue in the catalytic site to be important for regulation of kinase activity^49^. Nonetheless, this orientation occurs in all three of the high-resolution integrin β5-PAK4 structures (Fig. 4e). This unexpected component of PAK4 recognition of the integrin β5 cytoplasmic tail suggests insertion of a strongly electronegative residue towards the ATP cleft, and seems to be accommodated by the ATP-analogue moiety which shows poor electron density in all of the structures, perhaps due to low occupancy.

### Validation of the PAK4-β5 interface by mutagenesis

To validate the structurally-defined PAK4-β5 binding site we generated mutants in both PAK4 and integrin β5 and tested their effect in pull-down assays. Introduction of an R763E mutation in the β5 tail strongly impairs binding to PAK4-cat, and we hypothesize this is due to the loss of the salt bridge between Arg763 in β5 and Glu512 in PAK4. Interestingly, however, R765E mutation does not significantly impair binding, suggesting that Arg763 is critical for integrin binding (Fig. 4f). We mutated PAK4 residues Asp444 and Glu512 to disrupt integrin binding. These PAK4 residues are in the ‘substrate binding groove’ and their mutation is thought to deleteriously impact the ability of PAK4 to recognize downstream substrates. In pull-down assays with both the catalytic domain of PAK4 (Fig. 4g) and with full-length PAK4 (Fig. 4h), we observe strong inhibition of GFP-tagged PAK4-cat with D444A and E512R mutation. We found moderate effect with M482K mutation, which reached significance against the kinase domain, but not the full length, and we found no effect on binding for a nearby mutation in PAK4, Q358A. The interaction between PAK4 and integrin β5, therefore, seems to be driven by integrin β5 binding to the PAK4 substrate-binding site.

### β5 integrin inhibits PAK4 kinase activity

**Fig. 5:**
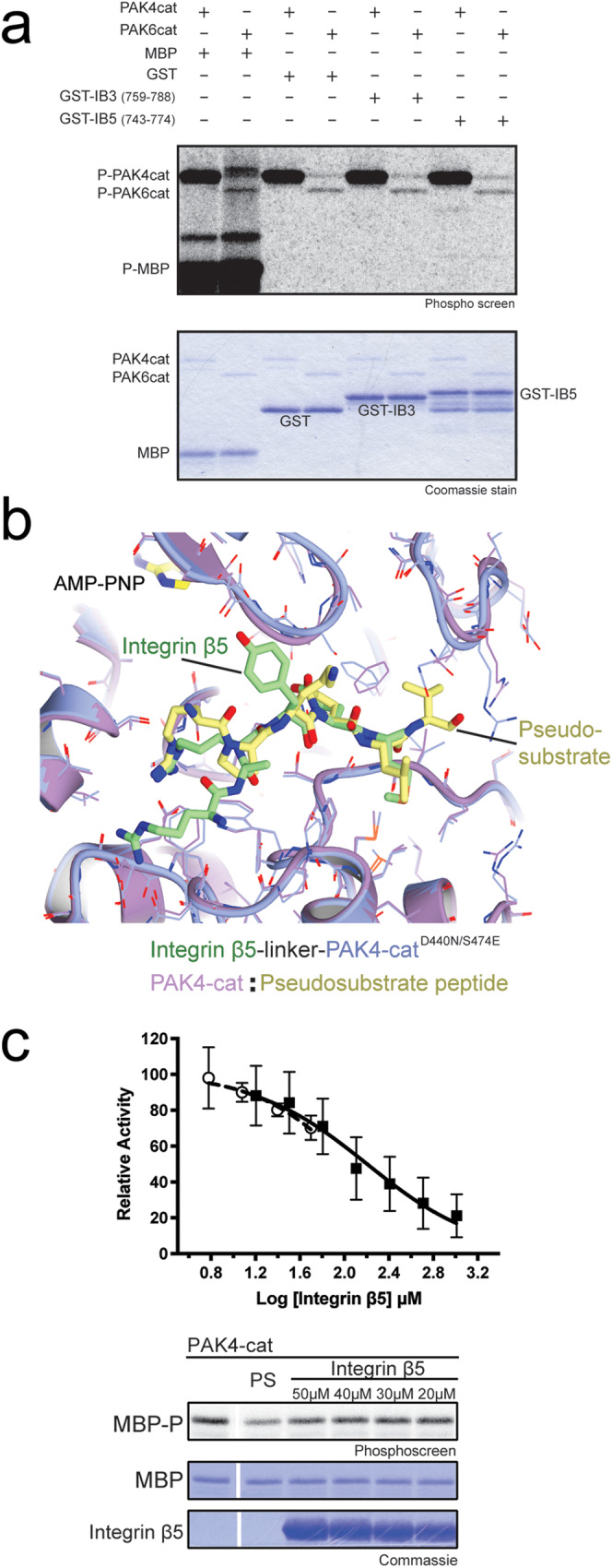
In vitro impact of integrin β5 cytoplasmic tail on PAK4 kinase activity. **a** PAK4 phosphorylates maltose binding protein and autophosphorylates itself, however, it shows little evidence of robust phosphorylation of phosphorylation of GST and a GST-fusion of integrin β5 encompassing residues 743–774. PAK4 also shows little evidence of robust phosphorylation of GST-integrin β3 (residues 759-788). Activity of PAK6 catalytic domain is shown for comparison. **b** Superposition of integrin β5 bound PAK4 with the structure of autoinhibitory pseudosubstrate bound PAK4cat (PDB: 4FII) showing a similar substrate binding groove is occupied. **c** Titration of full-length integrin β5 cytoplasmic tail suppresses PAK4cat phosphorylation of MBP (clear circles) with autoradiography and Coomassie stains shown. PS refers to 0.05 µM pseudosubstrate control peptide (RPKPLVDP)^25^. *N* = 3. Titration of 11mer integrin β5 peptide (ERSRARYEMAS) to inhibit phosphorylation of LIMK1 10mer peptide (RKKRYTVVGN) in the presence of 1 mg/ml BSA (black squares). *N* = 6. SD shown. Source data are provided as a Source Data file.

Based on the crystal structures and mutagenesis, PAK4 seems to bind integrin β5 in a non-catalytically competent conformation. This contrasts with previous reports which indicate that PAK4 is a kinase for integrin β5^35^, so we assessed the ability of PAK4 to phosphorylate integrin β5 in our hands. We find that kinase activity towards the full-length integrin β5 cytoplasmic tail was negligible and that the family member, PAK6, similarly does not display strong phosphorylation of integrin β5 (Fig. 5a).

We previously showed that PAK4 kinase activity is inhibited by an internal N-terminal pseudosubstrate sequence^25^. The PAK4 pseudosubstrate inhibition exhibits little sequence similarity with β5 tails but engages in a similar manner (Fig. 5b). We therefore wondered if integrin binding to PAK4 might suppress kinase activity. We tested this using in vitro kinase assays in the presence of full-length integrin β5 tail peptides and observed suppression of catalytic activity (Fig. 5c). We found that a short 11mer integrin β5 peptide can inhibit phosphorylation of a 10mer peptide corresponding to the phosphorylation site within LIM domain kinase 1, LIMK1 (Fig. 5c). Quantification of the inhibition reveals an IC50 of 160 ± 40 µM (Fig. 5c). The relatively weak inhibition of catalytic activity by β5 peptides is less pronounced than that seen with the N-terminal pseudosubstrate peptide^50^ but clearly demonstrates an impairment of PAK4’s ability to phosphorylate substrate in the presence of integrin β5, indicating that recruitment of PAK4 to integrin cytoplasmic tails suppresses PAK4 activity.

## Discussion

Integrin activity is modified by phosphorylation events^37,38^, but a number of protein kinases are also reported to bind to integrin cytoplasmic tails, in some cases directly through their kinase domains^34,39,51–54^. In this study, we provide molecular level evidence for how a kinase domain interacts with a cytoplasmic integrin tail. In our crystal structures we show that PAK4 is recruited to integrin β5 in a kinase-pseudosubstrate like manner, with the integrin engaging the kinase substrate binding site and suppressing kinase activity.

We determined four structures of PAK4 in complex with integrin β5. Our initial chimeras allowed us to both confirm that binding occurs in a 3.5 Å solution and to define the register of interaction in a structure that contained two PAK4 mutations of the catalytic Asp (D440N) and the activation loop phosphosite (S474E). These chimeric structures raised the interesting possibility that the complex between PAK4 and integrin β5 may be driven by a kinase-substrate like interaction, so we followed up by determining two further structures of the double mutant PAK4 (D440N/S474E) and of wild-type PAK4 in complex with the peptide sequence that we had mapped from the chimeras. The co-crystallization showed an almost identical interaction and revealed two insights.

First, pseudosubstrate regulation of PAK4 may be a broader aspect of modulation of this kinase group than previously thought. We previously showed that the autoinhibitory pseudosubstrate motif, centered around a proline residue (Pro50) is key for kinase modulation^25,50^, and other groups showed that binding partner proteins, INKA1 and INKA2, modulate activity by pseudosubstrate binding^30,31^. We then demonstrated that CDC42 binds to PAK4 using an extensive interface that includes a pseudosubstrate inhibition region^27^. Now, the addition of the PAK4-integrin β5 structures to this array further widens the potential modulation effects of kinase activity. Like most well studied PAK substrates^24,47,48^, in each of the pseudosubstrate interactions the recognition of the substrate seems to be driven by recognition of an arginine residue two or three amino acids N-terminal of the phosphoacceptor position, and perhaps this represents insight into the ability of kinase-pseudosubstrate complexes to mimic key features of kinase-substrate pairs, and illustrates that regulation of catalytic activity is influenced by many factors in the cell.

Second, the residue residing at the P-0 position, the site of phosphoacceptance in a canonical substrate, was in each of our structures a glutamic acid residue (integrin β5 residue Glu767). This unusual conformation was part of the driving force for us to determine co-crystal structures in addition to the chimera. We were concerned that the chimera, because of its replacement of the acidic Asp440 with Asn, was more able to accommodate a glutamic acid at this position than wild-type PAK4. Our two co-crystals of wild-type and D440N/S474E PAK4 mitigated these concerns by revealing almost identical integrin orientations, and almost identical placement of the glutamic acid. The insertion of an acidic residue into the catalytic cleft is unusual, but not unprecedented. The kinase CK1 binds to a phosphorylated substrate tightly as part of its regulation mechanism whereby the phosphoserine is located in the phosphoacceptor site, suggesting that anchor points between the bound substrate and the kinase facilitates the electrostatically-disfavored interaction^49^. In a similar way, the anchor points of the PAK4-integrin β5 interaction may favor complex formation over the unusual electrostatics of acidic residue insertion into the catalytic cleft.

**Fig. 6:**
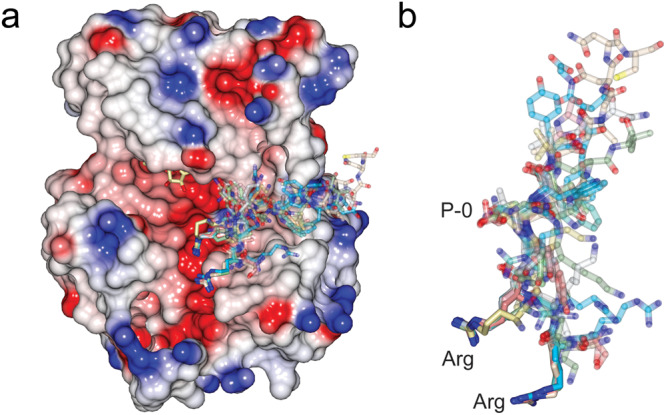
Comparison of PAK4 complexes. Superposition of PAK4 structures in complex with integrin β5 (7S46, 7S47, and 7S48), consensus peptide (2Q0N), PAKtide-S (4JDI)^47^, autoinhibitory pseudosubstrate (4FII)^25^, N-terminal polybasic region (5UPK)^27^, (Inka inhibitory pseudosubstrate (4XBU)^30^, LIMK1 substrate peptide (6WLY)^48^ and β-catenin substrate peptide (6WLX)^48^. **a** Electrostatic surface of PAKtide-S bound PAK4, sticks for bound AMP-PNP, and stick format peptides for each structure, when kinase domains are superposed on their C-lobes. **b** Close-up of the peptides, illustrating the similarity of location for the basic charged arginine residues. Phosphoacceptor site indicated as P-0.

Similarly, there have been multiple structures of the PAK4 previously determined in complex with substrates, pseudosubstrates, and binding partners. Intrinsic to each of these complexes is the interaction of the kinase domain through its substrate binding groove and partner short linear motifs. Each of the known binding partners contains basic charged residues which contact with the acidic patches within the kinase C-lobe, and their structural superposition (Fig. 6) suggests that the salt bridging of the arginine residues plays the key role in recognition and binding.

Previous literature has suggested a PAK4-integrin β5 interaction, but that the complex resulted in phosphorylation of integrin β5 and was mediated by the integrin S^759^ERS^762^ motif^34,35^. Although the SERS motif was originally thought to be important for interaction with PAK4, in our crystal structures only the final Ser762 is visible and is in weak electron density and not in a position available for catalysis. Furthermore, we see little evidence of robust integrin β5 phosphorylation by PAK4 in vitro, instead integrin β5 apparently weakly inhibits PAK4 phosphorylation of substrates. Thus, while our study supports the previous work describing a direct binding between PAK4 and integrin β5, by revealing the details of how this interaction is mediated and assessing consequences of binding on catalysis, it suggests that direct interaction between PAK4 and integrin cytoplasmic tails may suppress kinase activity of this key CDC42 effector.

## Methods

### Expression and purification

Expression and purification of PAK4 catalytic domain has been previously described^25,27,46–48^. Briefly, catalytic domain of PAK4 (UniProt ID:O96013) was sub-cloned into a modified pET28a vector (Novagen) with N-terminal hexa-histidine (6xHis) tag cleavable by Tobacco Etch Virus (TEV) protease. Integrin β5 residues 743–774 following four repeats of Ser-Ser-Gly linker were used to make chimeric PAK4 catalytic protein. The chimeric Integrin β5^743–774^-linker peptides(SSGSSGSSGSSG)-PAK4 catalytic domain with N-terminal hexa-histidine (6xHis) tag cleavable by thrombin was sub-cloned into a pET28a vector. Double mutated chimeric Integrin β5^743–774^-linker-PAK4cat at D440N and S474E was introduced using QuikChange (Agilent Technologies). The expression construct for the 6xHis-tagged β5 cytoplasmic tail was generated by PCR and subcloned into a pET32 vector.

PAK4 catalytic domain was expressed in BL21-CodonPlus(DE3)RILP (Agilent Technologies) cell and the chimeric protein was expressed in BL21(DE3)pLysS (Agilent Technologies) by induction with 0.5 mM isopropyl β-D-1-thiogalactopyranoside (IPTG) overnight at 18°C. Harvested pellets were suspended in lysis buffer (20 mM Tris-HCl (pH 8.0), 100 mM NaCl, 1 mM tris(2-carboxyethyl)phosphine (TCEP) and 0.1 mM phenylmethanesulfonylfluoride, PMSF) and lysed by sonication. The supernatants were affinity purified by HisTrap chelating column (GE) and then resolved over Resource Q (GE). Eluted PAK4-cat and chimeric protein was then loaded to a Superdex 200 Increase 10/300 GL (GE) column (20 mM Tris-HCl (pH 8.0), 0.15 M NaCl, 1 mM DTT). The 6xHis integrin β5 full-length cytosolic peptide (residues 743–799) was expressed in inclusion bodies in BL21(DE3) (Agilent Technologies) cells by induction with 0.5 mM IPTG overnight at 37 °C. After cell lysis, pellet was suspended with washing buffer (20 mM Tris-HCl (pH 8.0), 0.1 M NaCl, 1 mM TCEP, and 1% (*v*/*v*) Triton X-100) and then supernatant was discarded following centrifugation. 6xHis-integrin β5^743–799^ was then resuspended in 8 M urea-containing buffer (20 mM Tris-HCl pH 8.0, 0.1 M NaCl, 1 mM TCEP, and 8 M urea). The supernatant after centrifugation then was applied to Ni-NTA Agarose (Qiagen) to capture 6xHis tagged integrin β5^743–799^ peptide. On-column refolding was performed by reducing urea concentration gradually in washing buffer. Then eluted 6xHis integrin β5^743–799^ peptides and purified PAK4 catalytic domain were dialyzed against PBS buffer for ITC.

### Peptides

Integrin β5 peptide (760-Glu-Arg-Ser-Arg-Ala-Arg-Tyr-Glu-Met-Ala-Ser-770) for crystallization and LIM kinase 1 peptide (503-Arg-Lys-Lys-Arg-Tyr-Thr-Val-Val-Gly-Asn-512) were synthesized with 0.1 mM scale N-terminal acetyl and C-terminal amide and HPLC purified at Tufts University Core Facility.

### Crystallization and data collection

The chimeric integrin β5^743–774^-linker-PAK4cat protein was concentrated to 4 mg/ml in SEC buffer and incubated with 1 mM phosphoaminophosphonic acid-adenylate ester (AMP-PNP) and 5 mM MgCl_2_ prior to setting up crystallization drops at room temperature. The crystal of chimeric integrin β5^743–774^-linker-PAK4cat protein was crystallized in 100 mM sodium acetate pH 4.0, 0.7–0.8 M diammonium phosphate at room temperature. The crystals were cryoprotected with reservoir buffer and 30% (*v*/*v*) glycerol. Due to its low resolution (3.5 Å; see below) we decided to introduce double mutation at D440N and S474E in the chimeric protein. Another chimeric integrin β5^743–774^-linker-PAK4cat^D440N,S474E^ protein was incubated with 1 mM AMP-PNP and 5 mM MgCl_2_, and then crystallized in 0.1 M Tris-HCl (pH 8.5) and 0.5 M tri-sodium citrate buffer at room temperature. The crystals were cryoprotected with reservoir buffer plus 35 % (*v*/*v*) glycerol. Crystals were obtained using PAK4 at 4 mg/ml against 100 mM sodium acetate pH 4.0, 0.7–0.8 M diammonium phosphate at room temperature. Purified PAK4cat and double mutated PAK4cat^D440N,S474E^ proteins were incubated with 1 mM AMP-PNP and 5 mM MgCl_2_ in advance of crystallization trial with 11mer integrin β5 peptide^760–770^ dissolved in D/W. 2 mM integrin β5^760–770^ peptide was used for co-crystallization with PAK4cat protein, and 1 mM peptide was soaked into PAK4cat^D440N,S474E^ crystals. All data were collected at the Advanced Photon Source (APS) beamline 24-ID-E.

### Structure determination and refinement

Integrin β5^743–774^-linker-PAK4cat: Crystallographic data were processed to 3.5 Å resolution using the HKL 2000 package^55^. An initial molecular replacement solution was obtained using Phaser^56^ using the previously determined crystal structures of PAK4 catalytic domain (PDB ID:4FIF). This yield Z-score for translation functions of 10.4, and 1st round refinement with Refmac5^57^ yielded *R* and *R*_free_ values of 20.0 and 25.1 %. Clear positive electron density map around catalytic cleft was obtained, but due to poor resolution we decided to make inactive PAK4 domain by introducing D440N mutation in the chimeric protein. However, the inactive chimeric protein was insoluble presumably because of a lack of activation loop phosphorylation, so we additionally introduced the activation loop phospho-mimetic mutation, S474E. The integrin β5^743–774^-linker-PAK4cat^D440N,S474E^ data were processed to 2.0 Å using the HKL-2000 package, and initial phases generated using molecular replacement using PDB ID:4FIF as the model with Phaser yielding a translation function Z-score of 6.8. Refinement of the chimeric integrin β5^743–774^-linker-PAK4cat^D440N,S474E^ was conducted using Refmac5 with a maximum-likelihood target and TLS (translation, libration, screw). Model building was conducted in COOT^58^, and during refinement interpretable electron density for integrin β5 residues became clearly visible, which were built manually. Poor electron density is observed for adenine of AMP-PNP in integrin β5^743–774^-linker-PAK4cat^D440N,S474E^ structure. A total of 45 amino acids are not visible in the electron density to span a distance of ~21 Å for the cis complex, or a distance of ~31 Å for dimerization with symmetry-related molecule, so although the model was built in cis it is possible a trans complex may have crystallized. Model quality assessed using MolProbity^59^. Co-crystal structure complexes: Crystallographic data were processed to 1.9 and 2.1 Å using the HKL2000 package for PAK4cat- integrin β5^760–770^ peptide and PAK4cat^D440N, S474E^-integrin β5^760–770^ peptide, respectively. Initial phases were generated using Phaser with model PDB ID:4FIF and yielded translation Z-scores of 7.3 and 8.5 for PAK4cat- integrin β5^760–770^ peptide and PAK4cat^D440N, S474E^-integrin β5^760–770^ peptide. They were refined using Refmac5 with a maximum-likelihood target and TLS. Poor electron density is observed for adenine of AMP-PNP in both structures.

### Isothermal titration calorimetry

Purified PAK4cat was pre-incubated with 5 mM MgCl_2_ and 1 mM AMP-PNP and refolded integrin β5^743–799^ cytosolic peptides were all dialyzed against PBS buffer (pH 7.4) three times for the Nano-ITC (TA instrument) experiment. The reference cell was filled with water and the sample cell was filled with PAK4cat. Protein concentrations were determined using UV. For PAK4-integrin β5 interaction, 44 and 72 µM of PAK4cat were loaded in the sample cell and 407 µM of integrin β5^743–799^ peptides loaded in the syringe. ITC experiments were conducted at 25 °C using a stir speed of 350 rpm and incremental titration of 20 injections, 2.5 µL volume, and 300 s intervals. Buffer data (PBS into PBS titration) was subtracted from the corresponding experiment before data fitting. Raw data were processed and integrated with NanoAnalyze software (TA instruments). The first injection in each experiment was not considered for the analysis. Stoichiometry of interaction (*N*), dissociation constant (*K*_d_), enthalpy changes (Δ*H*), and entropy (Δ*S*) were determined using NanoAnalyze software.

### Kinase assays

Impact of full-length cytosolic integrin β5 on kinase activity was assessed using a radioactive assay with myelin basic protein (MBP) as substrate. Assays were performed by addition of 0.1 µM of PAK4cat kinase, 2 µM of MBP, cytosolic full-length integrin β5 ^743–799^ (50, 25, 12, and 6 μM), with 12 µM of cold ATP and 0.05 µCi of hot [γ33-P] ATP in Tris buffer (20 mM Tris-HCl (pH 8.0), 0.3 M NaCl, 1 mM TCEP, and 10 mM MgCl_2_) with a total volume of 25 µL. The reaction was conducted at 30 °C for 7–11 min then stopped by the addition of 5× sample buffer and analyzed by SDS-PAGE. Dried gels were analyzed by exposure to phosphor storage screen (GE Healthcare) followed by scanning using a Molecular Imager FX Pro Plus System (Bio-Rad) and quantified by optical densitometry Quantity One (Bio-Rad). Measurements were calculated from three independent experiments. Significant differences were calculated by Prism 7 (GraphPad Software) with ANOVA analysis. Impact of short integrin β5 peptide on kinase activity was assessed using a radioactive assay with a LIM kinase peptide (RKKRYTVVGN, LIMtide) as substrate. The concentration of PAK4 and LIMtide were kept constant at 0.2 and 50 µM, respectively. Increasing concentrations of integrin β5 peptide (ERSRARYEMAS) (0, 16, 32, 64, 128, 254, 512, 1024 µM) were titrated to the kinase-substrate mixture. The reaction was performed in the presence of 1 mg/ml BSA to mitigate non-specific binding. All components were reconstituted in kinase reaction buffer (20 mM HEPES pH 7.4, 10 mM MgCl_2_, 1 mM DTT). The reaction was initiated by adding ATP at a final concentration of 20 µM containing 0.05 µCi γ-^32^P ATP. The reaction mixture was incubated at 30 °C for 10 min, after which 7.5 µl aliquot was blotted onto P81 filter paper. The reaction was quenched by washing the P81 filter paper in 75 mM phosphoric acid three times with 5 min intervals. After a final wash with acetone, the filter paper was air dried and added to scintillation vials containing 6 ml optifluor scintillation fluid for analysis in scintillation counter. Duplicate measurements were calculated from three independent experiments. Prism 9 (GraphPad Software) was used for analysis.

### Integrin pull-down assays

Pull down assays were performed as previously described^45,60^. Briefly, constructs encoding an N-terminal His-tag followed by a helical region, a 4-glycine spacer, and an integrin cytoplasmic tail sequence were produced in BL21 (DE3) bacteria. Following bacterial cell lysis, recombinant protein was bound to Ni-NTA beads, eluted with imidazole, dialyzed and further purified by reverse-phase high-performance liquid chromatography (HPLC). Purified protein was lyophilized for storage and was used to coat fresh Ni-NTA beads to generate an affinity matrix for pull-down assays.

For pulldowns from cell lysates, human PAK4 constructs were generated in pEGFP (Takara Bio Inc.) by PCR amplification from a pLX304 PAK4 isoform 1 (Uniprot O96013-1) construct kindly provided by Michael Calderwood (Dana-Farber Cancer Institute). Mutations and deletions were introduced by QuikChange mutagenesis or PCR amplification. Chinese hamster ovary (CHO) cells were seeded on 10 cm tissue culture dishes and transiently transfected with GFP-tagged constructs using polyethylenimine (Polysciences). After 24 h, cells were harvested and lysed in buffer X (1 mM NaVO4, 50 mM NaF, 40 mM sodium pyrophosphate, 50 mM NaCl, 150 mM sucrose, 10 mM Pipes, pH 6.8) containing 0.5% Triton X-100, 0.2% deoxycholic acid, and complete EDTA-free protease inhibitor mixture (Roche, Indianapolis, IN, USA) for 15 min at 4 °C. After clarification by centrifugation, lysate was diluted in buffer X-T (buffer X with 0.05% Triton X-100) and incubated with His-tagged integrin tails coupled to Ni-NTA beads for 4 h with rocking at 4 °C. For experiments using purified PAK4cat, protein was incubated with His-tagged integrin tails coupled to Ni-NTA beads in buffer X-T for 2 h with rocking at 4 °C. Beads were then washed three times with buffer X-T, and bound proteins were fractionated by reducing SDS-PAGE and analyzed by immunoblotting with anti-GFP antibodies (600-101-215, Rockland). Immunoblots were imaged on an Odyssey IR imaging system (Li-Cor; Lincoln, NE, USA) and analyzed using Image Studio Lite (Li-Cor; Lincoln, NE, USA). For the quantification of binding from immunoblots, the fluorescence intensity of the band corresponding to bound material was quantified as a fraction of the fluorescence of the input material band for each condition. Loading of integrin tails was assessed by Coomassie Blue staining. Data were analyzed using GraphPad Prism.

### Statistics and reproducibility

Mean ±s.d. or s.e.m. values are indicated and were derived using Prism (GraphPad Software). Statistical significance was determined using one-way analysis of variance (ANOVA), followed by Dunnett’s multiple comparison test performed using Prism Software. *P* values <0.05 were considered significant for all analyses (*P < 0.05, **P < 0.001, ****P < 0.0001). Reproducibility of results and sample sizes are discussed in the figure legends. Data from all ITC experiments are shown in supplemental material.

### Reporting summary

Further information on research design is available in the Nature Research Reporting Summary linked to this article.

## Supplementary information

Supplementary Information

Description of Additional Supplementary Files

Supplementary Data 1

Reporting Summary

## Data Availability

Crystallographic coordinates and structure factors have been deposited in the Protein Data Bank under accession codes: 7S46, 7S47, and 7S48. X-ray diffraction images are available online at SBGrid Data Bank^61^: 10.15785/SBGRID/852 (PDB ID: 7S46), 10.15785/SBGRID/854 (PDB ID: 7S47) and 10.15785/SBGRID/853 (PDB ID: 7S48). Uncropped gels are shown in Supplementary Fig. 2 and source data shown in Supplementary Data 1.
