# Author Correction: Molecular basis for integrin adhesion receptor binding to p21-activated kinase 4 (PAK4)

**DOI:** 10.1038/s42003-023-05176-4

**Published:** 2023-07-31

**Authors:** Byung Hak Ha, Sezin Yigit, Nalini Natarajan, Elizabeth M. Morse, David A. Calderwood, Titus J. Boggon

**Affiliations:** 1 The Department of Pharmacology, Yale University, 333 Cedar St., New Haven, CT 06520 USA; 2 The Department of Cell Biology, Yale University, 333 Cedar St., New Haven, CT 06520 USA; 3 The Department of Molecular Biophysics and Biochemistry, Yale University, 333 Cedar St., New Haven, CT 06520 USA

**Keywords:** X-ray crystallography, Kinases

Correction to: *Communications Biology* 10.1038/s42003-022-04157-3, published online 17 November 2022.

In the Acknowledgements section, the National Institutes of Health grant to authors D.A.C. and T.J.B. should have read “R01GM138411”. The original article has been corrected.

