# Supplementary material for: Molecular basis for integrin adhesion receptor binding to p21-activated kinase 4 (PAK4)
Source: Commun Biol. 2022 Nov 17;5:1257. doi: 10.1038/s42003-022-04157-3 (PMC9669019; doi:10.1038/s42003-022-04157-3)

Supplementary Information for:

Title

Molecular basis for integrin adhesion receptor binding to p21-activated kinase 4 (PAK4)

Authors

Byung Hak Ha[1], Sezin Yigit[1], Nalini Natarajan[1], Elizabeth M. Morse[2], David A. Calderwood[1,2]*, and Titus J. Boggon[1,3]*

Affiliation

The Department of Pharmacology[1], Yale University, 333 Cedar St., New Haven, CT 06520, USA, the Department of Cell Biology[2], Yale University, 333 Cedar St., New Haven, CT 06520, USA, and of the Department of Molecular Biophysics and Biochemistry[3], Yale University, 333 Cedar St., New Haven, CT 06520, USA

Supplementary Table 1. Thermodynamic properties of the interaction between PAK4cat and integrin β5 cytoplasmic tail.

Sample Cell	Syringe	K_d (μM)	N	ΔH (kJ/mol)	ΔS (J/molK)
PAK4cat	Integrin $\beta5^{743\text{-}799}$	5.1	1.4	-7.7	75.7
PAK4cat	Integrin $\beta5^{743\text{-}799}$	4.8	0.7	-9.2	70.8

Supplementary Table 2. RMSD difference table for between PAK4 catalytic domains.

	$\beta5^{743-774}$ -linker-PAK4cat	$\beta5^{743-774}$ -linker-PAK4cat^{D440N,S474E}	PAK4cat: $\beta5^{760-770}$	PAK4cat^{D440N,S474E} : $\beta5^{760-770}$
$\beta5^{743-774}$ -linker-PAK4cat		1.1 Å (290 Cα)	1.0 Å (290 Cα)	1.0 Å (290 Cα)
$\beta5^{743-774}$ -linker-PAK4cat^{D440N,S474E}			0.3 Å (290 Cα)	0.2 Å (290 Cα)
PAK4cat: $\beta5^{760-770}$				0.2 Å (290 Cα)

Supplementary Table 3. RMSD difference table for between integrin β5 peptides.

	$\beta5^{743\text{-}774}$ -linker-PAK4cat^{D440N,S474E}	PAK4cat: $\beta5^{760\text{-}770}$	PAK4cat^{D440N,S474E}: $\beta5^{760\text{-}770}$
$\beta5^{743\text{-}774}$ -linker-PAK4cat^{D440N,S474E}		0.49 Å (6 Cα)	0.14 Å (6 Cα)
PAK4cat: $\beta5^{760\text{-}770}$			0.38 Å (7 Cα)

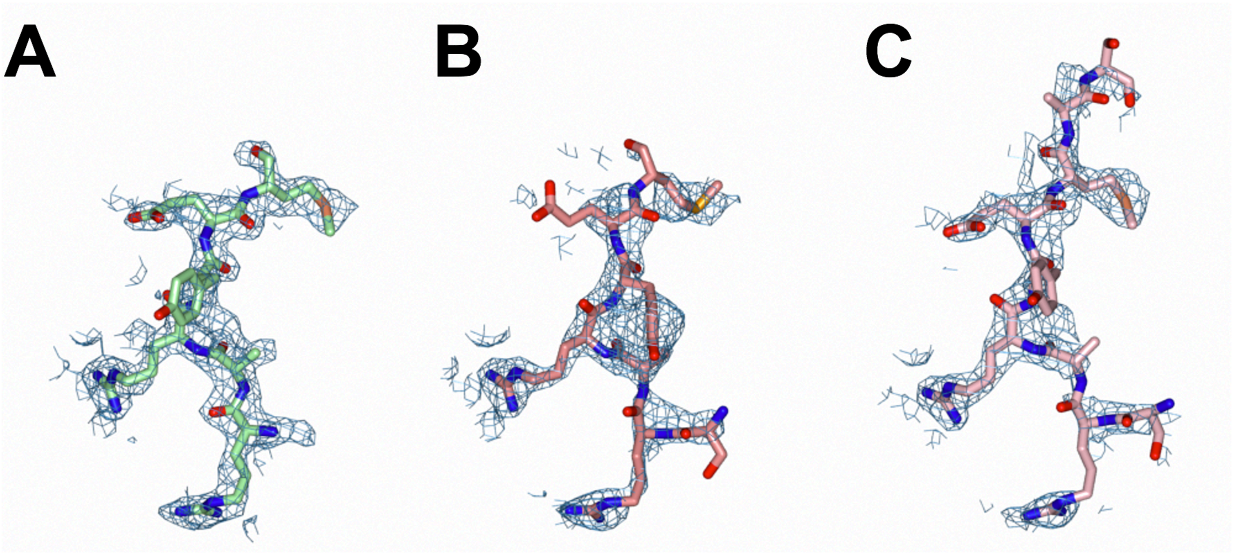

Supplementary Figure 1. Electron density for integrin β5. 2Fo-Fc electron density maps contoured at 1-sigma for **A)** integrin β5-PAK4$^{D440N/S474E}$ chimera (PDB ID: 7S47). **B)** PAK4 co-crystallized with integrin β5 peptide -Glu^{760}-Arg-Ser-Arg-Ala-Arg-Tyr-Glu-Met-Ala-Ser^{770}- (PDB ID: 7S48). **C)** D440N/S474E PAK4 co-crystallized with integrin β5 peptide -Glu^{760}-Arg-Ser-Arg-Ala-Arg-Tyr-Glu-Met-Ala-Ser^{770}- (PDB ID: 7S46).

Supplementary Figure 2. Uncropped gels. Uncropped gels for indicated figures are shown.

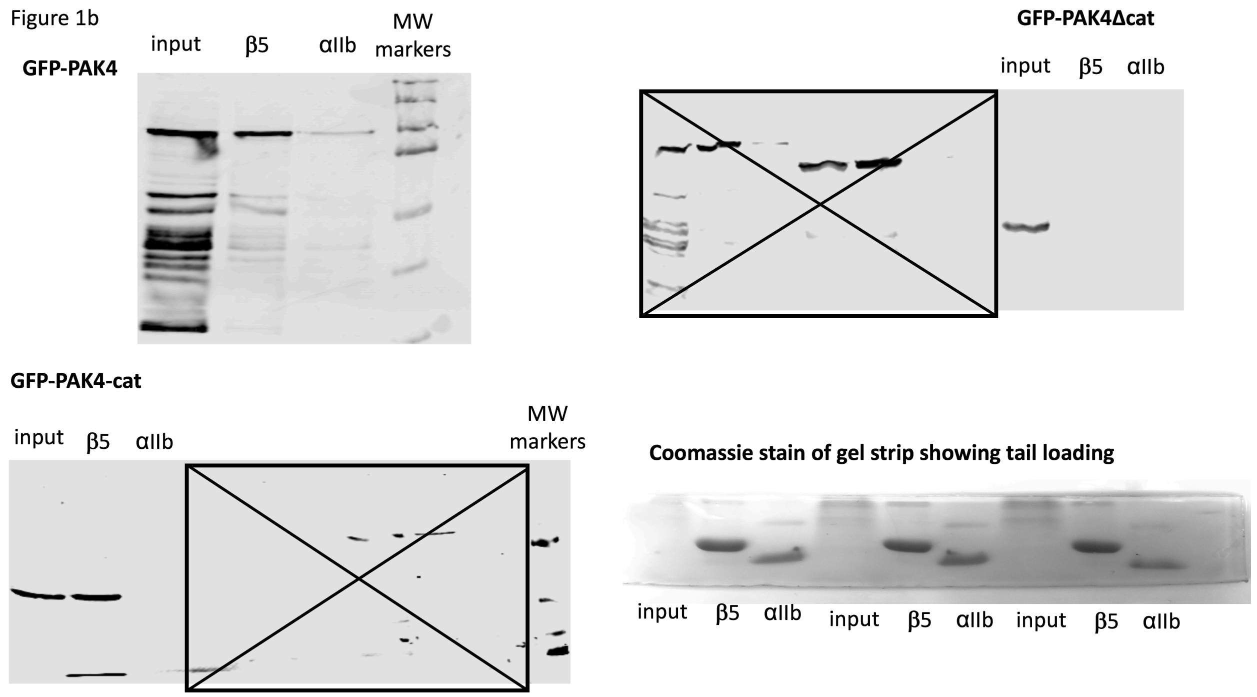

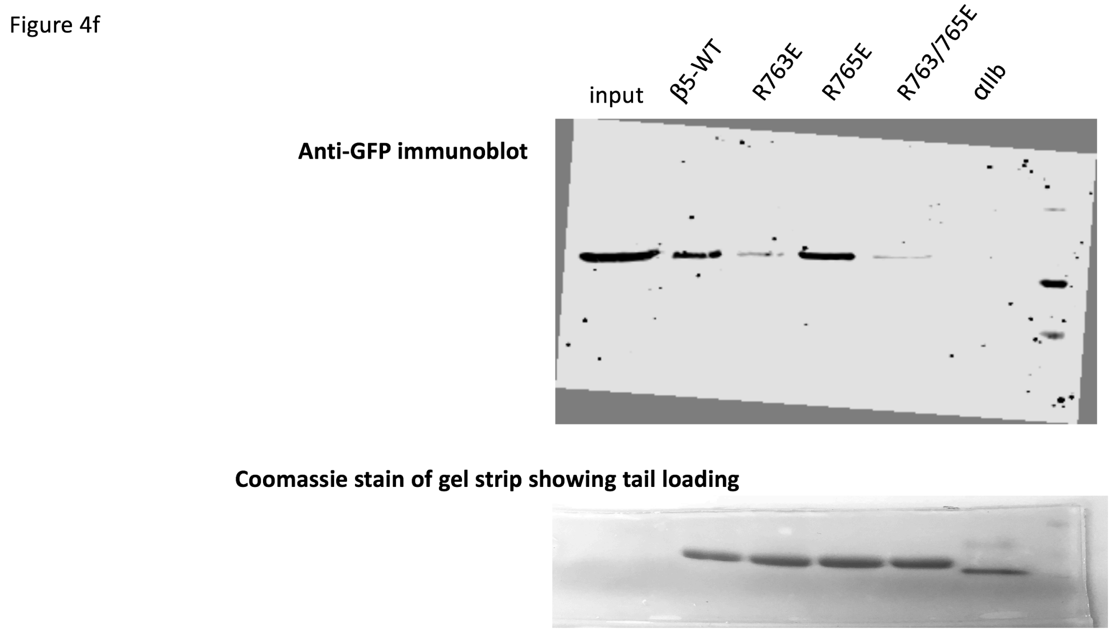

Figure 4g

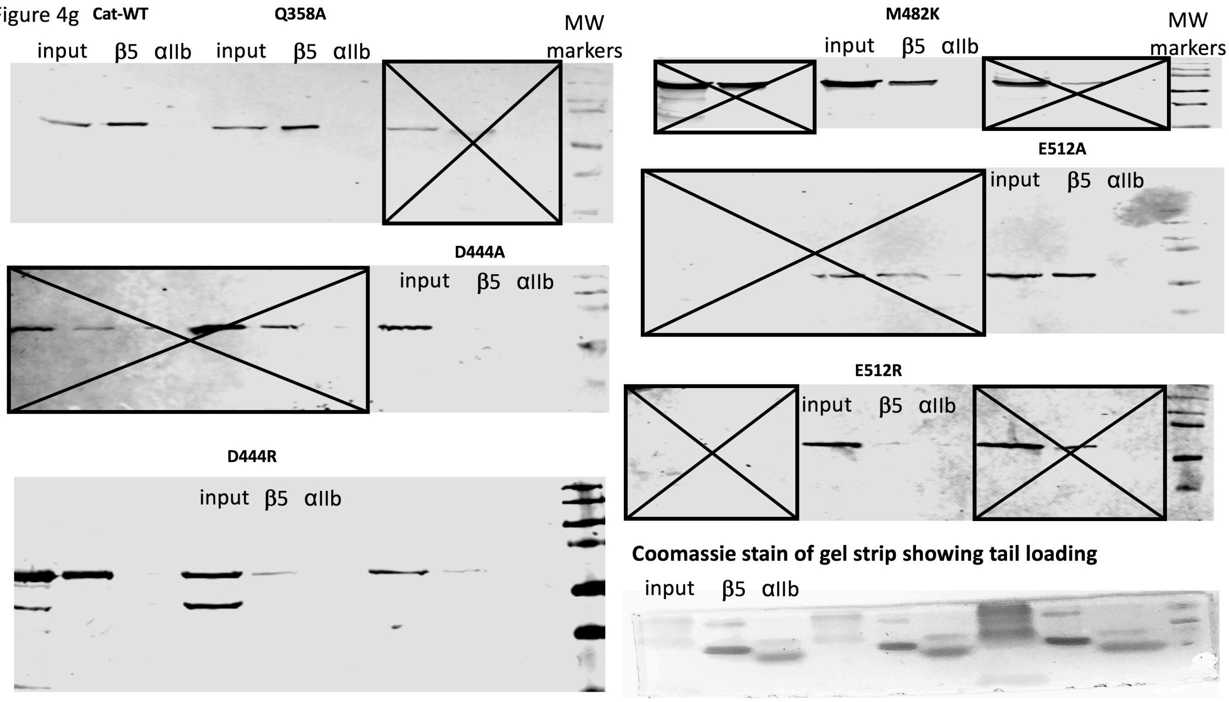

Figure 4h

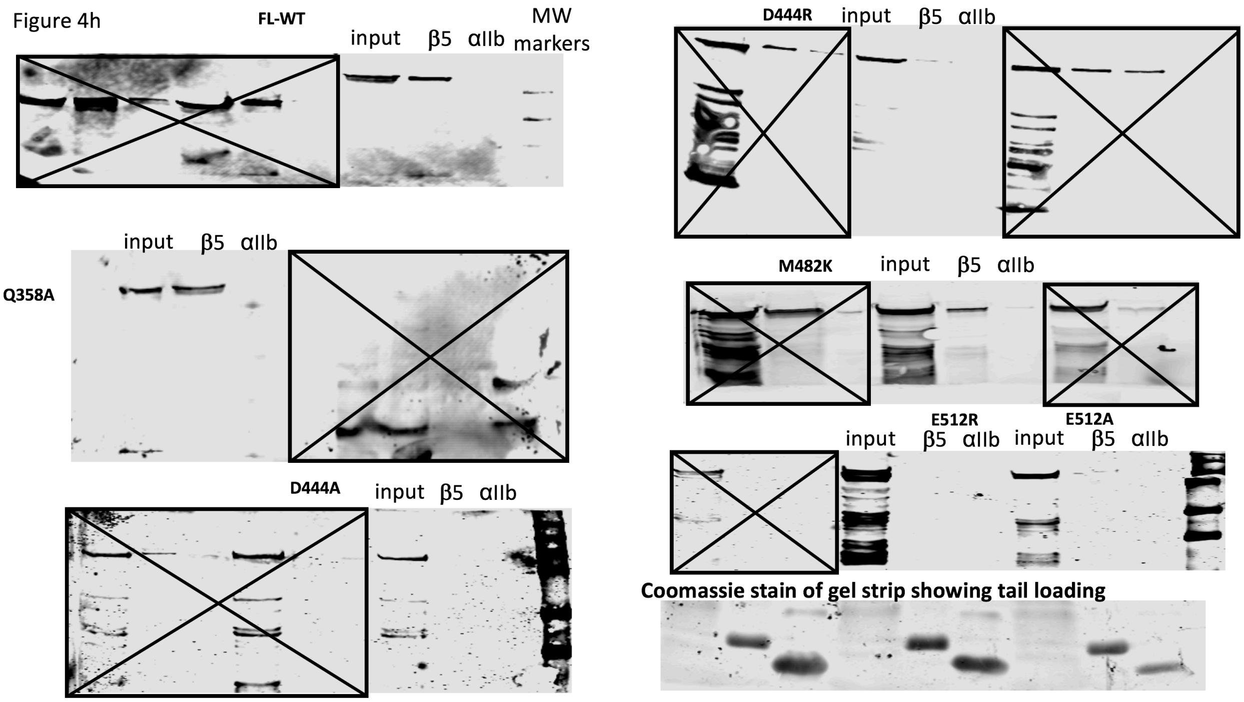

Figure 5a

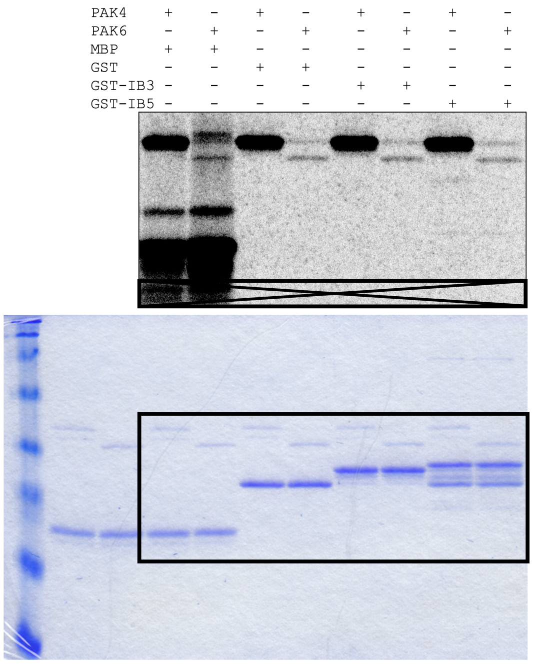

Figure 5c

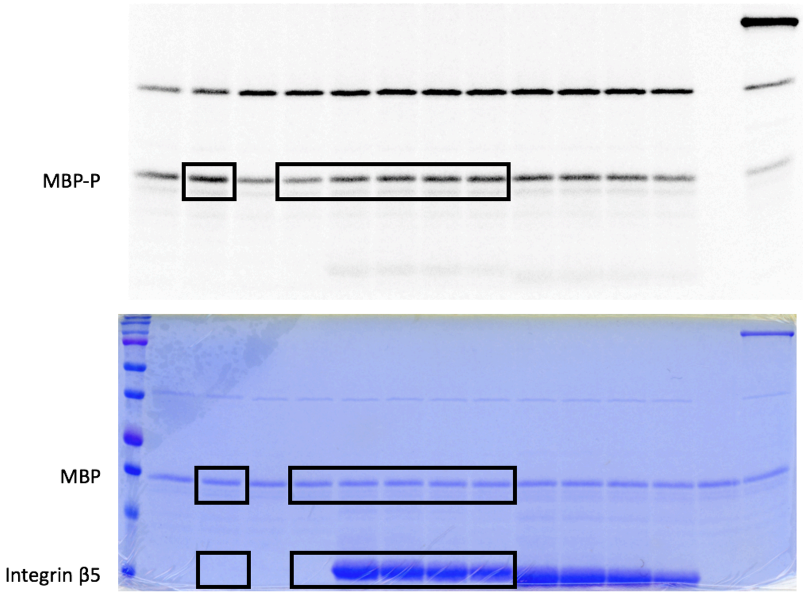

Supplement: Supplementary file 1 — Supplementary Information [file 42003_2022_4157_MOESM1_ESM.pdf]
