# Supplementary material for: Molecular basis for integrin adhesion receptor binding to p21-activated kinase 4 (PAK4)
Source: Commun Biol. 2022 Nov 17;5:1257. doi: 10.1038/s42003-022-04157-3 (PMC9669019; doi:10.1038/s42003-022-04157-3)
Supplement: Supplementary file 2 — Description of Additional Supplementary Files [file 42003_2022_4157_MOESM2_ESM.pdf]

Description of Additional Supplementary Files

File name: Supplementary Data 1
Description: Source Data for figures 1c, 1d, 1f, 4g, 4h and 5c
